# Brimonidine Therapy for Protection From Noise‐Induced Hearing Loss

**DOI:** 10.1111/acel.70481

**Published:** 2026-04-09

**Authors:** Jing Cai, Na Zhang, Yongdong Song, Chengfang Chen, Ligang Kong, Yu Ai, Yu Jin, Qian Gao, Daogong Zhang, Lei Xu, Haibo Wang

**Affiliations:** ^1^ Department of Otolaryngology‐Head and Neck Surgery, Shandong Provincial ENT Hospital Shandong University Jinan Shandong China

**Keywords:** aging, brimonidine, ear, noise, spiral ganglion neurons, synapses

## Abstract

Noise exposure is a known cause of hearing loss, and only a few effective preventive drugs are available. Therefore, in this study, we aimed to investigate the protective effects of brimonidine on noise‐induced inner ear hearing impairment in mice and explore its underlying mechanisms and long‐term outcomes. Mice were randomly divided into control, noise exposure, and brimonidine groups. A 62‐week follow‐up was conducted after noise exposure. Brimonidine inhibited the noise‐induced increase in inner ear glutamate concentration and downregulated inflammatory factors and immunoglobulins. Brimonidine decreased glutaminase and VGLUT2/3 expression and reduced glutamate synthesis and vesicle transport without affecting its clearance, thereby decreasing glutamate excitotoxicity and protecting synapses and spiral ganglion neurons long term. Mice exposed to noise could temporarily restore their hearing thresholds; however, their auditory function in old age remained significantly worse than those that received brimonidine‐mediated cochlear protection in youth. These findings highlight the importance of enhancing noise protection from an early age.

## Graphical abstract

In this study, we demonstrate that brimonidine protects against noise‐induced hearing loss in young mice by suppressing glutamate synthesis and vesicle transport, thereby reducing excitotoxicity and preserving synaptic integrity. Such early intervention may prevent age‐related hearing loss, highlighting brimonidine's potential as a therapeutic agent for acoustic trauma.

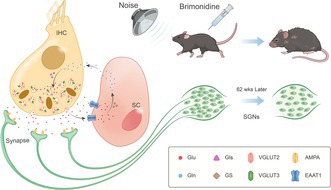

## 1 Introduction

Noise‐induced hearing loss (NIHL) remains a major public health problem worldwide (Chadha et al. 2021). Hearing loss caused by acute noise exposure (NE) is classified as a temporary threshold shift (TTS) or permanent threshold shift based on NE intensity (Wang et al. 2025). In common TTS, hearing recovers to normal levels within a few weeks. However, rapid, extensive, and irreversible loss of ribbon synapses between spiral ganglion neurons (SGNs) and inner hair cells (IHCs) occurs after NE, resulting in delayed and progressive SGNs loss several months later (Kujawa and Liberman 2009, 2015; Lin et al. 2011). Relatively normal hearing patterns in the early stages of injury mask widespread synaptic lesions, as verified in animal experiments (Kujawa and Liberman 2009). This “hidden hearing loss” warrants early intervention. Evidence from studies on noise‐induced deafness in young people indicates that NE in early life can lead to hearing loss later in life (Balk et al. 2023; Kujawa and Liberman 2006). Therefore, protecting synapses during the early stage of injury to reduce subsequent loss of SGNs is important for preventing acquired sensorineural hearing loss.

The mechanisms of NE‐induced damage include excitotoxicity, mechanical injury, oxidative stress, calcium ion overload, and immune responses, among which glutamate‐mediated excitotoxicity is the most important (Kim et al. 2019; Moser and Starr 2016; Paciello et al. 2021; Wu et al. 2022). Glutamate is the principal excitatory neurotransmitter at the synapse between cochlear hair cells and the axons of auditory neurons. During NE, excessive glutamate release and impaired clearance from the synaptic cleft cause calcium overload, mitochondrial dysfunction, and neuronal edema or loss (Liberman and Kujawa 2017).

Potential treatment options for NIHL include exogenous antioxidants, glutamate antagonists, calcium antagonists, neurotrophic factors, and glucocorticoids (Han et al. 2015; Hashimoto et al. 2019; Hu et al. 2020; Khan et al. 2000; Min et al. 2023; Yu et al. 2024). These agents can mitigate the impact of NE through various mechanisms; however, their effects on preventing or restoring hearing are limited. The U.S. Food and Drug Administration has not approved any pharmacological interventions for NIHL (Le Prell 2022). Hearing aids and cochlear implants are currently used as alternative treatments for sensorineural hearing loss. These devices can improve hearing to some extent; however, they do not fully restore the function of damaged inner ear cells and neurons (Carlson 2020). Therefore, it is essential to investigate the pathogenesis of NIHL and explore potential therapeutic strategies that target its underlying causes.

Brimonidine, a first‐line drug for the treatment of glaucoma, is a highly selective α_2_‐adrenergic receptor (α_2_‐AR) agonist that lowers intraocular pressure and protects the optic nerve (Maciulaitiene et al. 2024). It exerts significant protective effects by reducing cyclic adenosine monophosphate (cAMP) levels, inhibiting voltage‐gated Ca^2+^ channels, regulating the β‐amyloid pathway, upregulating neurotrophic factors, and alleviating glutamate‐mediated excitotoxicity in neurons (Jung et al. 2015; Lee et al. 2012; Nizari et al. 2016; Wang et al. 2024). Our previous study reported that brimonidine protects SGNs cultured in vitro from glutamate damage via an anti‐apoptotic mechanism (Cai et al. 2013). However, it remains unclear whether brimonidine exerts a neuroprotective effect on inner ear hair cells and auditory nerves following NE. Therefore, we aimed to investigate the short‐ and long‐term protective effects of brimonidine on SGN synaptic structures and its underlying mechanisms of action.

## 2 Methods

### 2.1 Animals and Grouping

Eight‐week‐old C57BL/6J mice were obtained from the Animal Center of Shandong University (Jinan, China). The mice were housed in a controlled environment (temperature: 20°C–22°C; humidity: 40%–70%; 12/12‐h light/dark cycle) with ad libitum access to food and water.

**FIGURE 1:**
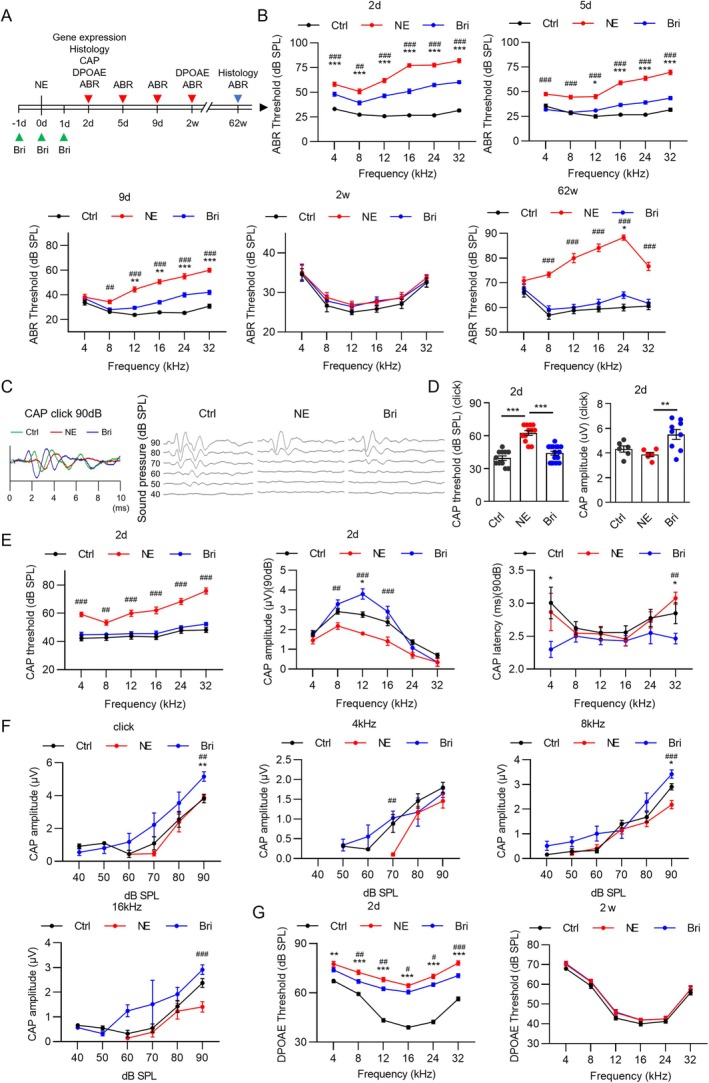
Brimonidine attenuates noise‐induced hearing loss in adult mice. (A) Administration and testing time points. (B) ABR results of three groups of mice at different time points. Hearing loss in the brimonidine group was consistently less severe than that in the NE group. Although hearing in the NE group recovered to baseline levels by 2 weeks post‐exposure, ABR thresholds in this group significantly increased again by 62 weeks post‐exposure, whereas the hearing level in the brimonidine group did not differ from that in the control group. (C) CAP waveform diagrams of the three groups. (D) Threshold and amplitude of CAP under click at 90 dB. (E) CAP threshold, amplitude, and latency for each frequency. (F) CAP amplitude of click, 4, 8, and 16 kHz. (G) DPOAE values. *n* = 6–26 for ABR. *n* = 8–15 for DPOAE. The ABR and DPOAE measurements were obtained from the same mice; the CAP threshold was from a separate group. Statistical analysis: One‐way ANOVA, *p* < 0.05, compared with control (*) and NE (#) groups. Symbols 1, 2, and 3 indicate *p* < 0.05, 0.01, and 0.001, respectively.

Ninety C57BL/6J mice were randomly divided into three groups (*n* = 30 per group): NE (+NE, −Bri), brimonidine (+NE, +Bri), and control (−NE, −Bri). Mice in the NE group were exposed to noise ranging from 8 to 16 kHz at an intensity of 100 dB for 2 h. Mice in the brimonidine group received intraperitoneal (IP) injections of brimonidine (1 mg/kg, UK‐14,304, Sigma‐Aldrich) 1 day before NE, on the day of NE, and on the first day post‐exposure. Mice in the control group received an equivalent volume of saline via IP injection following the same regimen. On the second day after NE, mice in each group underwent auditory brainstem response (ABR), compound action potential (CAP), and distortion product otoacoustic emission (DPOAE) testing and were subsequently euthanized. Cochlear RNA sequencing was performed in 17 mice, and molecular biology experiments were conducted in the remaining animals. The remaining mice in each group underwent ABR testing at 2 weeks post‐exposure and were then raised until 62 weeks post‐exposure (*n* = 8). At 62 weeks, the mice underwent ABR and histologic testing before euthanasia (Figure 1A).

To assess whether brimonidine itself impacts hearing and synapses, an additional 12 mice were randomly assigned to two groups: sham (−NE, −Bri) and single brimonidine (−NE, +Bri). Mice in the single brimonidine group received IP injections of brimonidine (1 mg/kg, *n* = 6) for 3 consecutive days, whereas mice in the sham group received IP saline injections for 3 days.

### 2.2 Ethics Statement

All research protocols were approved by the Animal Care Committee of Shandong University (No. ECAESDUSM 20,123,011). All experiments were conducted in accordance with ARRIVE guidelines and complied with the Guide for the Care and Use of Laboratory Animals of the National Institutes of Health. Only male C57BL/6J mice were used in this study; therefore, the sex‐based influence on the results was not evaluated.

### 2.3 NE

Awake and unrestrained mice were exposed to a 100 dB sound pressure level (SPL; 8–16 kHz) octave‐band noise stimulus for 2 h using a sound generator (Crown CDi1000, AVIS). Noise calibration was performed before each exposure session. Notably, the sound pressure levels varied by 1 dB between the cages. Mice in the brimonidine group were exposed to noise 0.5 h after IP brimonidine administration.

### 2.4 Auditory Function Tests

Auditory function was assessed using ABR, DPOAE, and CAP tests. Briefly, the mice were anesthetized with xylazine (6 mg/kg) and ketamine (50 mg/kg). ABRs were recorded using subdermal needle electrodes (vertex‐ipsilateral ear‐back position). Furthermore, the recording electrode was placed in the round window niche for cochlear nerve CAP. TDT System III (Tucker‐Davis Technologies) produced click and tone burst stimuli at frequencies of 4, 8, 12, 16, 24, and 32 kHz, each with a duration of 10 ms and rise/fall time of 0.5 ms. Electrode responses were amplified, filtered, and averaged (1024 samples for ABR and 512 for CAP; alternating polarities). Specifically, the initial stimulation intensity was set to 90 dB SPL and decreased in steps of 5 dB until the response was undetectable. The lowest intensity that elicited a response was recorded as the ABR threshold. The intensity that triggered a CAP amplitude of 3 μV was defined as the CAP threshold. CAP amplitude, measured as the voltage difference between the first negative peak and subsequent positive peak, was plotted against the stimulus intensity to produce an input/output function at each tested frequency. DPOAEs were recorded using primary tones with a frequency ratio of 1.2, with the level of f2 maintained at least 10 dB lower than that of the f1 level, while incrementing both in steps of 5 dB. The sound pressure within the ear canal was amplified and digitally sampled, and fast Fourier transforms were computed and averaged using both the waveform and spectral averaging methods. The 2f1‐f2 DPOAE amplitude and surrounding noise floor were extracted from the data. DPOAE threshold was defined as the f1 level required to elicit a response of ˗5 dB SPL.

### 2.5 Sample Collection

Cochlear tissues were harvested, and a portion was processed for transmission electron microscopy (TEM). Another portion was fixed overnight at 4°C with 4% paraformaldehyde (PFA; Sigma‐Aldrich) and rinsed with phosphate‐buffered saline (PBS; pH 7.4) to remove residual PFA. Thereafter, the samples were either prepared for frozen sectioning or subjected to microdissection. For immunofluorescence (IF) staining, cochlear basilar membrane (BM) tissues were placed on small glass slides pre‐coated with Cell‐Tak.

### 2.6 Synaptic Ribbon Counts

We longitudinally sectioned the modiolus and examined three segments of the basilar membrane. We further segmented the target frequency range in each turn, with each segment approximately 1 mm long. Cochlear frequency mapping identified basal (80%–90% from apex, 40–50 kHz), middle (40%–60%, 16–24 kHz), and apical (10%–20%, 9–12 kHz) turns (Manalo et al. 2020) (Francis et al. 2004). A 63× oil immersion objective with 3.5× digital zoom was used to capture whole‐mount images. Each image included at least five complete cells. We manually counted the total number of paired and unpaired synaptic ribbons based on C‐terminal binding protein 2 (Ctbp2, green) staining in each IHC.

### 2.7 Frozen Sections

Briefly, the cochleae were fixed overnight at 4°C in 4% PFA, decalcified in PBS containing 10% ethylenediaminetetraacetic acid (Solarbio) at 4°C for 24 h, dehydrated through sequential incubations in PBS supplemented with 10%, 20%, and 30% sucrose, and embedded in optimal cutting temperature compound (Tissue‐Tek, Sakura Fine‐Tek). Thereafter, the samples were cut into 7‐μm sections using a cryostat (Leica CM1850, Leica).

### 2.8 IF

Cochlear BM tissues and frozen sections were permeabilized with 0.5% Triton X‐100 (Sigma‐Aldrich) for 1 h and blocked with 1% fish gelatin solution (G7765; Sigma‐Aldrich) for 1 h. Thereafter, the samples were incubated overnight at 4°C with specific primary antibodies (Table S1) diluted in 1% PBS‐BSA solution. After washing with PBS, the samples were incubated in the dark with FITC‐ or TRITC‐conjugated donkey anti‐rabbit, anti‐mouse, or anti‐goat secondary antibodies (Table S1) and DAPI (D9542, Sigma‐Aldrich) diluted in 1% BSA‐PBS (1:1000) at 37°C for 1 h. After washing with PBS, the coverslips were mounted, and images were obtained using a laser‐scanning confocal microscope (Leica SP8; Leica).

### 2.9 TEM

Briefly, cochlear BM tissues were collected, rinsed with PBS, and immediately placed in a fixative solution containing 2% PFA and 2.5% glutaraldehyde (Sigma‐Aldrich, G5882; pH 7.4). Thereafter, the samples were dehydrated and embedded in Epon 812 (45345; Sigma‐Aldrich). Semi‐thin sections were stained with toluidine blue, and ultrathin sections were stained with lead citrate and uranyl acetate. Finally, the samples were examined using TEM (JEOL‐1200EX, JEOL‐1010) at Shandong Weiya Laboratory, and images were acquired using a Morada‐G2 CCD camera.

### 2.10 Enzyme‐Linked Immunosorbent Assay

Briefly, the inner ear tissue of the mice was harvested, snap‐frozen in liquid nitrogen, and stored at −80°C until analysis. Thereafter, the samples were lysed in a buffer and homogenized. After centrifugation of the homogenate, the supernatant was collected for enzyme‐linked immunosorbent assay (ELISA). Enzymes and standard glutamate (ab83389; Abcam) were used to determine glutamate concentrations in the inner ear according to the manufacturer's instructions. A microplate reader (Bio‐Rad) was used to detect signals at 450 nm.

### 2.11 RNA Sequencing Analysis

Total RNA was extracted from the cochleae of 17 mice (three control, seven NE, and seven brimonidine) using TRIzol reagent (Thermo Fisher Scientific) according to the manufacturer's instructions. RNA quantity and purity were assessed using a Bioanalyzer 2100 and RNA 6000 Nano LabChip Kit (Agilent). High‐quality RNA samples (RIN > 7.0) were used to construct sequencing libraries. Therefore, the transcriptomes of the three groups were analyzed using an Illumina NovaSeq 6000 platform (Shandong Xiuyue Biotechnology Co. Ltd.). Differential expression analysis was performed to identify differentially expressed genes (DEGs) between the three groups using DESeq2 software with the following thresholds: false discovery rate < 0.05 and absolute fold change ≥ 2. Kyoto Encyclopedia of Genes and Genomes (KEGG) pathway analyses were conducted for DEGs.

### 2.12 RNA Extraction and qPCR

Total RNA was extracted from BM tissues and purified using TRIzol reagent (Invitrogen). One portion was used for exploratory RNA sequencing analysis, whereas the other was independently preserved for validation experiments. Complementary DNA (cDNA) was synthesized via reverse transcription using the RevertAid First Strand cDNA Synthesis Kit (Thermo Fisher Scientific), according to the manufacturer's protocol. Quantitative real‐time PCR (qRT‐PCR) was performed using SYBR Premix Ex Taq (Takara Bio). GAPDH was used as the reference gene. Data analysis was performed using the Eppendorf Realplex 2 system. Primer sequences used for amplification are listed in Table S2.

### 2.13 Western Blot Analysis

Total protein was extracted from cochlear BM tissues using cold RIPA lysis buffer supplemented with a protease inhibitor cocktail. Thereafter, equal amounts of protein samples were heat‐denatured at 99°C for 10 min, separated using 10% SDS‐PAGE gel electrophoresis, and transferred onto polyvinylidene difluoride membranes (Millipore). After blocking with 5% skim milk for 1 h, the membranes were incubated with specific primary antibodies at 4°C for 15 h (Table S1). The following day, the membranes were incubated with horseradish peroxidase‐conjugated secondary antibodies for 1 h. Protein bands were visualized using an enhanced chemiluminescence kit (Millipore). All blocking, incubation, and washing steps were performed using Tris‐buffered saline with 0.05% Tween 20.

### 2.14 Image Analysis

Confocal images of ribbon synapses were acquired using a Leica SPE confocal microscope and analyzed using ImageJ software to measure ribbon synapse diameters. We defined any immunostaining spots exceeding the mean ± 2 × standard deviation (SD) in each group as abnormal. In frozen sections, SGNs with large round nuclei were counted within a complete Rosenthal's canal. For each experiment, at least six samples were analyzed to obtain the average value for each group.

### 2.15 Statistics and Reproducibility

All statistical analyses were performed using SPSS version 25.0. One‐way analysis of variance was used to assess differences among multiple groups, and unpaired *t*‐tests were used to compare two groups. Data are presented as mean ± SD. All experiments were repeated at least three times. Statistical significance was set at *p* < 0.05.

## 3 Results

### 3.1 Brimonidine Alleviates NIHL in Adult Mice and Impacts Long‐Term Hearing

In this study, NE significantly impaired auditory sensitivity. At 2 days post‐exposure, the ABR thresholds increased significantly at all frequencies (*p* < 0.001), with an increase of approximately 45 dB at 16–32 kHz. Hearing function gradually returned to normal levels. Brimonidine protected hearing thresholds in noise‐exposed mice (*p* < 0.05). Specifically, temporary hearing loss of approximately 20 dB was observed; however, hearing returned to normal 2 weeks post‐exposure (Figure 1B). Temporary restoration of hearing function occurred after NE; however, ABR thresholds in the NE group increased significantly at 62 weeks post‐exposure, whereas those in the brimonidine group decreased significantly (Figure 1B). In the absence of noise exposure, brimonidine alone did not affect hearing thresholds in mice (Figure S1A).

NE significantly increased the CAP threshold at various frequencies. Brimonidine administration significantly attenuated the NE‐induced increase in CAP threshold. NE significantly decreased the amplitude at 90 dB and 8–16 kHz; however, brimonidine reversed this decrease (Figure 1E,F). Notably, this finding was confirmed at 80 dB and 12 kHz (Figure S2A), indicating that NE damaged the synapses and that brimonidine provided some protection. At 90 dB, brimonidine significantly shortened CAP latency at 32 kHz (Figure 1E). Similarly, latency was significantly shortened at 80 dB and 32 kHz (Figure S2B). Latency reflects the conduction time of the auditory nerves.

To assess the function of outer hair cells (OHCs) in the cochlea, we measured DPOAE thresholds. NE increased the DPOAE thresholds compared with the baseline levels at 2 days post‐exposure; however, brimonidine administration reduced the thresholds at 8, 12, 24, and 32 kHz. DPOAE thresholds were normalized in both the brimonidine and NE groups at 2 weeks post‐exposure (Figure 1G). Overall, these results were consistent with those of ABR, suggesting that brimonidine attenuates NIHL in adult mice and that the normal function of hair cells correlates with their preserved quantity.

### 3.2 Brimonidine Reduces Synaptic Loss and Long‐Term SGN Loss After NE

**FIGURE 2:**
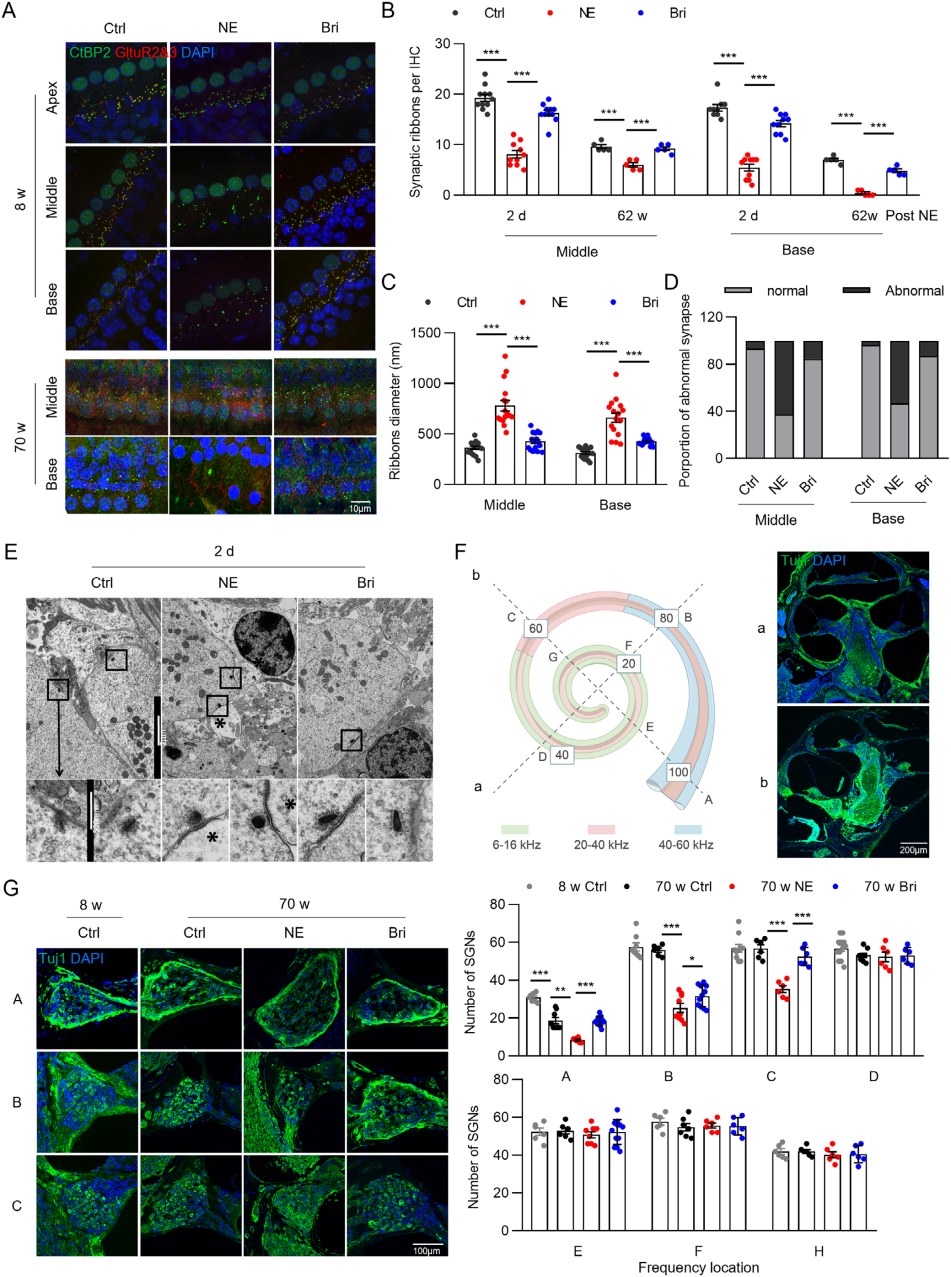
Brimonidine protects synapses and SGNs. (A) Representative images of IHC synapses in three groups at 2 days and 62 weeks post‐exposure. (B) Quantification of synapse numbers. *n* = 5–11 IHCs from 3 mice. (C) Quantification of synapse diameters. *n* = 16 ribbon synapses from 3 mice. (D) Quantification of abnormal synapse numbers. *n* = 3. (E) Synaptic ultrastructure in each group under TEM, * indicates neuronal edema. (F) The modiolar region was cut into two perpendicular sections (lines a and b), and the number of SGNs at the corresponding positions in each group was counted. (G) In representative frequency sections of basal and middle turns, the number of SGNs in the three groups of 70‐week‐old mice was reduced compared to that at 8 weeks, particularly in the NE group; brimonidine reversed SGN. *n* = 6–12 sections from 3 mice. *p < 0.05, **p < 0.01, ***p < 0.001.

IF staining for Ctbp2 (green) and glutamate receptor 2/3 (red) was performed on the BM tissue to observe the synaptic zone and AMPA‐type receptors. IHCs and OHCs were preserved (data not shown), and the average number of synapses per IHC was counted. NE significantly reduced synaptic survival (Figure 2A,B). At 2 days post‐exposure, synapse counts in the basal and middle turns decreased significantly from 17.33 ± 2 and 19.27 ± 2.28 to 5.45 ± 2.19 (69% decrease) and 8.1 ± 2.33 (58% decrease), respectively. In the brimonidine group, synapse counts in the basal and middle turns decreased by 18% (from 17.33 ± 2 to 14.2 ± 1.99) and 16% (from 19.27 ± 2.28 to 16.27 ± 1.79), respectively. In the NE group, synapse counts in the basal turn further decreased from 17.33 ± 2 to 0.4 ± 0.55 and were almost undetectable in 70‐week‐old mice. Synapse counts in the control group decreased significantly at 70 weeks; however, 40% of basal turn synapses remained (from 17.33 ± 2 to 7 ± 0.71), whereas 28% remained in the brimonidine group (from 17.33 ± 2 to 4.8 ± 0.84). The middle region had more synapses than the basal region. At 70 weeks, synapse counts in the three groups decreased by 50% (19.27 ± 2.28 to 9.6 ± 0.89, control), 69% (19.27 ± 2.28 to 6 ± 1, NE), and 52% (19.27 ± 2.28 to 9.2 ± 0.84, brimonidine), respectively. No significant differences were observed in apical turn synapse counts among the three groups at 8 weeks (Figure S3); however, data on apical turn synapses in aged mice are lacking. Overall, these results indicate that brimonidine has a significant protective effect on synapses and confirm that the basal turn of the cochlea is more fragile than that of the middle turn.

Furthermore, we observed significant changes in synaptic morphology. In the NE group, several synapses were abnormally increased (Figure 2C) and became misaligned with the IHC membrane, suggesting that they may no longer contribute to effective synaptic transmission. Notably, the number of normal‐sized synapses in the basal and middle turns did not exceed 50% and 40%, respectively (Figure 2D). In contrast, the number of normal‐sized synapses in both turns exceeded 80% in the brimonidine group, with synapses showing uniform size and orderly arrangement (Figure 2D). In the absence of noise exposure, brimonidine alone did not affect synapse number in mice (Figure S1B,C).

TEM revealed that the postsynaptic components of auditory nerve terminals were extensively swollen and vacuolated in the NE group at 2 days post‐exposure, with reduced electron density. The synaptic ribbon structures remained largely intact, although their shape occasionally changed from an elliptical to a circular shape. In contrast, synaptic morphology appeared normal, with no significantly swollen nerve terminals in the brimonidine‐treated group (Figure 2E).

In addition, we counted the number of SGNs in each group. No differences were observed in the number of SGNs among the three groups at 2 days post‐exposure (results not shown). Notably, the number of SGNs in the control group decreased at the 60 kHz position (from 30.86 ± 2.27 to 18.75 ± 4.8) at 70 weeks compared with that in young mice, indicating that aging resulted in approximately 39% neuronal loss (Figure 2G). Similarly, the number of SGNs in the NE group decreased rapidly (from 30.86 ± 2.27 to 8.33 ± 1.21), with approximately 73% neuronal loss at 60 kHz. However, brimonidine treatment reversed NE‐induced SGN loss, with approximately 40% neuronal loss (from 30.86 ± 2.27 to 18.42 ± 2.39) (Figure 2G). At the 40 kHz position, SGN counts in the control, NE, and brimonidine groups decreased by 3% (from 57.63 ± 6.07 to 55.83 ± 2.79), 56% (from 57.63 ± 6.07 to 25.33 ± 6.87), and 45% (from 57.63 ± 6.07 to 31.73 ± 5.62), respectively. At the 30 kHz position, the decrease was smaller: SGN counts in the control, NE, and brimonidine groups decreased by 0.4% (from 56.9 ± 6.57 to 56.67 ± 5.05), 38% (from 56.9 ± 6.57 to 35.33 ± 4.41), and 8% (from 56.9 ± 6.57 to 52.5 ± 4.89), respectively (Figure 2G). At lower frequencies, including the middle‐to‐apex turns, SGN counts remained unchanged. Overall, neuronal and synaptic loss patterns were consistent.

### 3.3 Brimonidine Can Suppress Glutamate Accumulation

**FIGURE 3:**
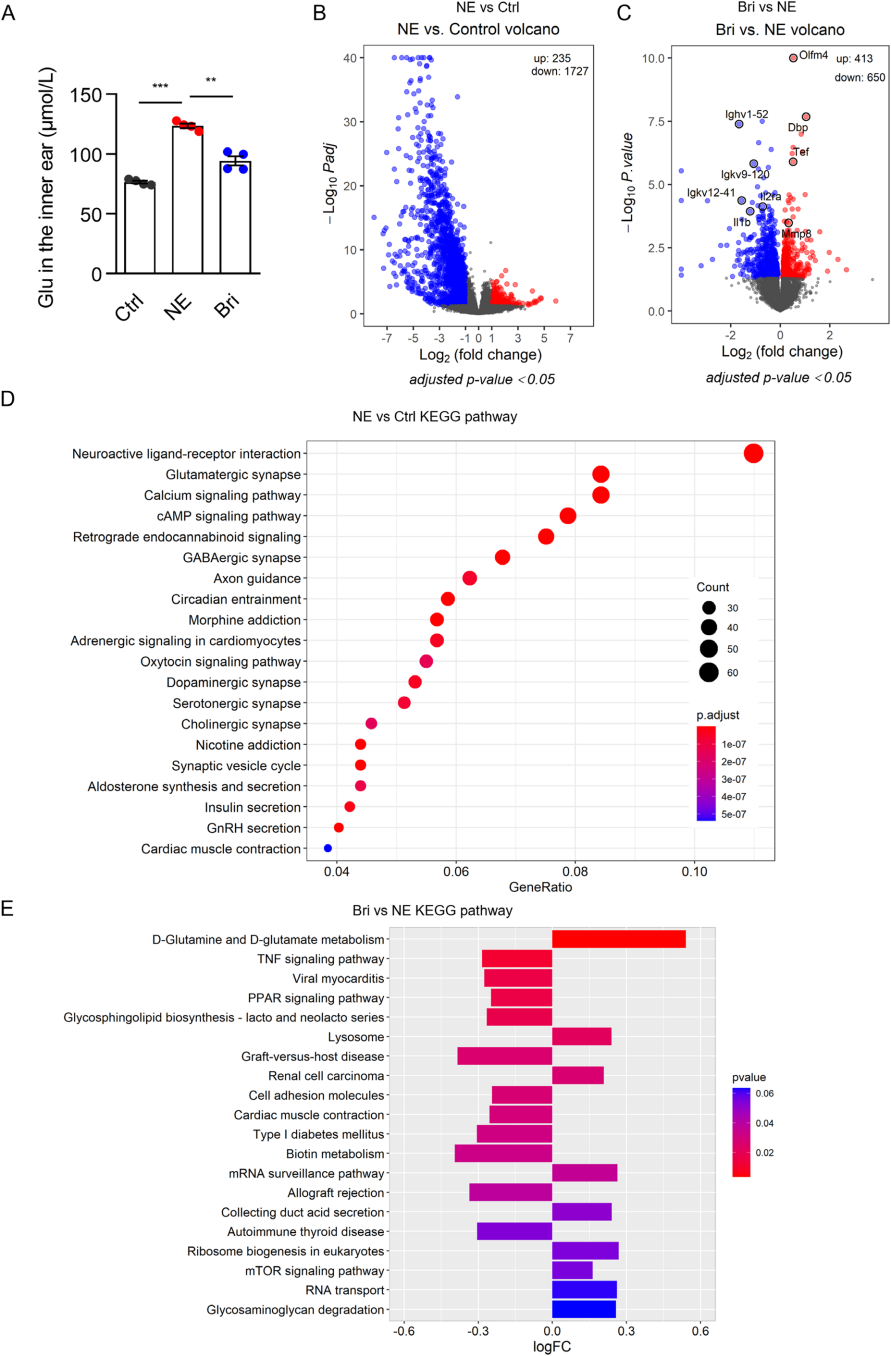
Transcriptomic analysis of the three groups. (A) Glutamate (Glu) concentration (*n* = 4). *p < 0.05, **p < 0.01, ***p < 0.001. (B, C) Volcano plot showing the differentially expressed genes (DEGs). Genes with adjusted *p*‐value < 0.05 and fold change (FC) ≥ 2 or FC ≤ 0.5 are considered as DEGs. (D) KEGG enrichment analysis of the DEGs in NE mice compared with the control group. (E) GSVA KEGG enrichment analysis of all expressed genes in NE mice compared with the brimonidine group. *n* = 3–7. The bar chart shows the 20 representative enriched pathways.

The primary mechanism underlying NE‐induced damage is glutamate excitotoxicity. To assess whether brimonidine reduced glutamate accumulation in the cochlea, whole cochleae were collected 2 days post‐exposure, and glutamate content was measured using an ELISA. Expectedly, glutamate content in the cochlea was significantly higher (*p* < 0.001) in the NE group than in the control group (123.55 ± 3.6 ng/g vs. 76.43 ± 2.25 ng/g; Figure 3A). However, brimonidine treatment significantly reduced glutamate levels (94.2 ± 7.58 ng/g; *p* < 0.01) compared with the NE group (Figure 3A). Collectively, these results indicate that brimonidine protects against NE‐induced hearing loss, partly by mediating glutamate accumulation in the cochlea.

### 3.4 Brimonidine Affects the Glutamate–Glutamine Cycle in the Synaptic Cleft of IHCs

**FIGURE 4:**
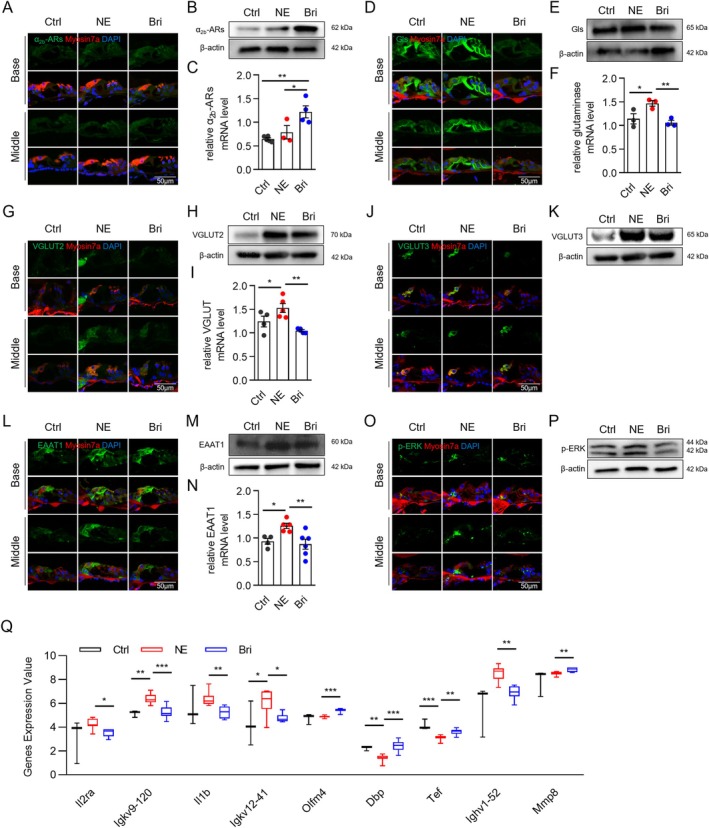
Brimonidine reduces glutamate synthesis and transport and decreases p‐ERK expression. (A–C) Expression of α_2b_‐AR was significantly increased in the brimonidine group. (D–N) Brimonidine influences the glutamate–glutamine cycle; it decreases the expression of glutaminase. (D–F) VGLUT2 (G–I), VGLUT3 (J–K), and EAAT1 (L–N) induced by NE (O–P). Brimonidine reversed the NE‐induced increase in p‐ERK expression. *n* = 5. *p < 0.05, **p < 0.01, ***p < 0.001. (Q) Nine differentially expressed genes were identified between the brimonidine and NE groups.

To investigate the protective mechanism of brimonidine, cochleae from the three groups were collected 2 days post‐exposure for RNA sequencing. Genes with an adjusted *p*‐value < 0.05 and a fold change ≥ 2 or ≤ 0.5 were defined as DEGs. Compared with the control group, 1962 genes were differentially expressed in the NE group, including 235 upregulated and 1727 downregulated genes (Figure 3B). In the brimonidine group, 1063 genes were differentially expressed compared with the NE group, including 413 upregulated and 650 downregulated genes (Figure 3C). KEGG enrichment analysis revealed that “Neuroactive ligand–receptor interaction,” “Glutamatergic synapse,” “Calcium signaling pathway,” and “cAMP signaling pathway” were significantly enriched in the NE group compared with the control group (Figure 3D). In addition, gene set variation analysis (GSVA) KEGG analysis was performed on all expressed genes in the brimonidine and NE groups. The top‐ranked pathway in the brimonidine group was “D‐glutamine and D‐glutamate metabolism” (Figure 3E). Therefore, we focused on the key factors involved in the glutamate–glutamine cycle. IF, qPCR, and western blotting showed that glutaminase (GLS), VGLUT 2/3, and EAAT1 levels increased significantly (Figure 4A–P), suggesting an increase in glutamate synthesis, transport, and clearance. Brimonidine reversed this trend (*p* < 0.05), indicating that brimonidine reduced glutamate synthesis and transport. VGLUT1 levels decreased in the NE and brimonidine groups, whereas glutamine synthetase levels remained unchanged (Figure S4G–K).

### 3.5 Brimonidine Alleviates Inner Ear Inflammation and Reduces the Expression of p‐ERK

RNA sequencing revealed that NE increased the levels of key inflammatory regulators (interleukin‐1β [*IL‐1β*] and *IL‐2ra*) and immunoglobulins (*Ighv1‐52*, *Igkv9‐120*, and *Igkv12‐41*). However, brimonidine significantly reduced the levels of inflammatory regulators and immunoglobulins (Figure 4Q; Figure S5), suggesting that it can counteract the inflammatory effects of NE in the cochlea. NE downregulated the circadian rhythm–regulating factor *Dbp*; however, brimonidine treatment prevented this change. Brimonidine upregulated *mmp8*, *tef*, and the anti‐apoptotic factor *Olfm4* (Figure 4Q; Figure S5). In addition, brimonidine treatment effectively reversed NE‐induced upregulation of p‐ERK expression (Figure 4O,P).

## 4 Discussion

**FIGURE 5:**
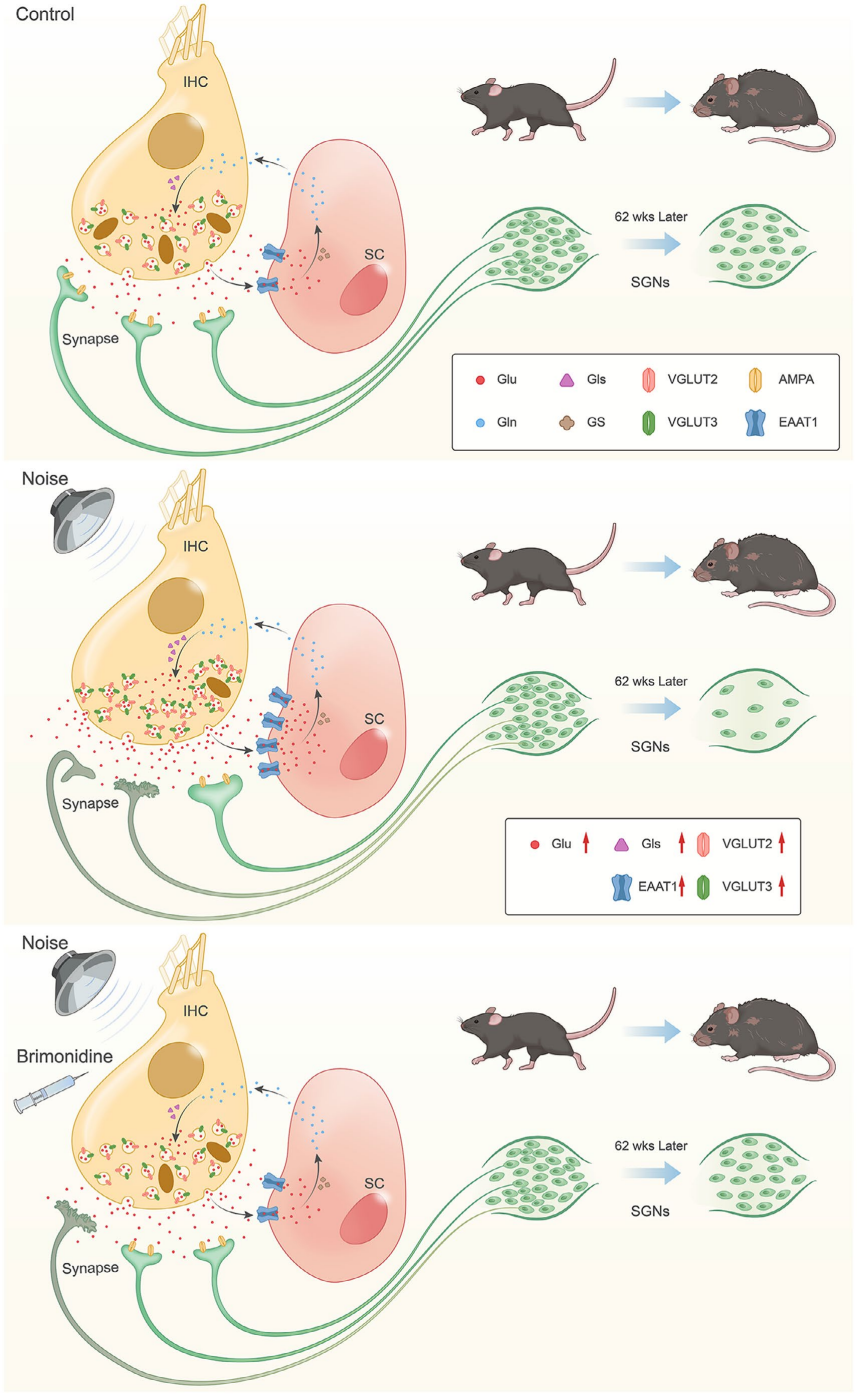
Schematic representation of glutamate–glutamine circulation in the cochlear IHC synaptic cleft and brimonidine protective mechanism. Brimonidine downregulated NE‐induced increases in GLS and VGLUT2/3, reduced glutamate concentration, protected the synapse, and ultimately preserved SGNs in the long term. Glu, glutamate; GS, glutamine synthetase; GLS, glutaminase; Gln, glutamine.

Over the past decade, extensive research has been conducted on the treatment and prevention of NE‐induced hearing loss (Han et al. 2015; Hashimoto et al. 2019; Min et al. 2023; Yu et al. 2024). Mechanistically, NE induces excitotoxic damage mediated by glutamate release and inflammatory responses (Munzel et al. 2017). We reported that brimonidine reduced glutamate levels in animals with cochlear NE, downregulated the levels of inflammatory factors (IL‐1β and IL‐2ra), and suppressed the expression of immunoglobulins. Furthermore, brimonidine protected the postsynaptic region, resulting in a reduction in delayed neuronal loss (Figure 5). Specifically, hearing gradually returned to normal levels in young mice. However, hearing was significantly better in the brimonidine group than in the NE group at 62 weeks post‐exposure. Overall, these findings suggest that noise exposure accelerates age‐related hearing loss (Shehabi et al. 2022) (Kujawa and Liberman 2006) (Fernandez et al. 2015) (Wu et al. 2021), underscoring the importance of early protection against NE.

The GSVA KEGG pathway analysis showed significant differences in the enrichment of glutamate metabolism‐related pathways between the brimonidine and NE groups. Activation of the α_2_‐AR can reduce glutamate release at the presynaptic site (Dong et al. 2008; Laudenbach et al. 2002). A previous study reported that α_2_‐AR selectively inhibits excitatory transmission mediated by AMPA glutamate receptors by downregulating cAMP and PKA (Hayashida et al. 2018). Brimonidine treatment effectively reversed NE‐induced increase in glutamate level. In IHCs, the primary source of glutamate is glutamine, and GLS mediates its conversion to glutamate (Oguchi et al. 2012). Brimonidine significantly inhibited the increase in GLS levels, indicating suppression of glutamate synthesis. Glutamate‐mediated synaptic transmission requires VGLUTs to load glutamate into synaptic vesicles. VGLUT3 is the most critical transporter, and IHCs in VGLUT3‐deficient mice lack glutamate release (Kim et al. 2019). Brimonidine reversed the increase in VGLUT2/3 expression, indicating that brimonidine inhibited glutamate transport. EAAT1 (GLAST), a significant glutamate clearance protein, inhibits extracellular glutamate accumulation (Glowatzki et al. 2006). In this study, NE upregulated EAAT1 expression, indicating that the inner ear increased EAAT1 expression to prevent glutamate‐induced toxicity. However, brimonidine treatment effectively downregulated EAAT1 expression, possibly by decreasing local glutamate concentrations. Overall, brimonidine modulates the glutamate–glutamine cycle at the synaptic cleft, reducing glutamate concentration by suppressing its synthesis rather than enhancing its clearance.

The inner ear possesses a robust immune system. Immune and inflammatory responses in the cochlea occur within 1–2 days after NE, peaking at 3–7 days (Wood and Zuo 2017). Yang et al. observed an increase in the expression of immune and inflammatory factors in the cochlea after NE using high‐throughput sequencing (Yang et al. 2015). Regulating these responses may positively influence the development of NIHL (Maeda et al. 2018). Our sequencing results revealed that NE increased the expression of *IL‐1β, IL‐2ra*, and immunoglobulins, including *Ighv1‐52, Igkv9‐120*, and *Igkv12‐41*. In contrast, brimonidine significantly downregulated inflammatory factors and immunoglobulins, indicating that brimonidine may counteract the inflammatory effects of NE in the cochlea.

ERK phosphorylation can trigger an immune response in the cochlea and may help recruit activated macrophages to damaged areas (Herranen et al. 2018). In the present study, ERK was activated in response to acoustic damage, primarily in the basal supporting cells adjacent to hair cells. However, the role of ERK activation in the inner ear after acoustic trauma remains controversial (Arthur et al. 2006; Lahne and Gale 2008). Akiyama et al. reported that the inhibition of ERK1/2 activation can significantly alleviate nerve cell damage (Kano et al. 2008). In the present study, brimonidine inhibited NE‐induced p‐ERK1/2 production, consistent with trends observed in previous in vitro experiments (Cai et al. 2013). Collectively, these results suggest that reducing p‐ERK activity may be one of the mechanisms by which brimonidine protects synapses.

A recent study of human cochleae from older individuals reported significant age‐dependent degeneration of SGNs, with approximately 30% neuronal loss in advanced age (Makary et al. 2011). In addition, the rate of SGN loss in older animals ranges from 40% to 50% (Kujawa and Liberman 2009, 2015; Lin et al. 2011). The absence of ribbon synapses and afferent terminals has a long‐term impact on the survival of afferent neurons; however, the status of synapses in elderly animals exposed to noise remains unknown. Conclusive evidence regarding noise‐induced pathological changes in cochlear synapses in humans is limited (Bramhall et al. 2019) (Shehabi et al. 2022). Our mouse model data suggest that neuronal and synaptic counts, particularly in the basal turn, were significantly lower in 70‐week‐old mice than in younger animals. NE exacerbated age‐related neuronal and synaptic loss, whereas brimonidine attenuated these changes and effectively protected synapses and neurons. Consistent with previous reports (Kujawa and Liberman 2009), synaptic and neuronal loss were consistent, mainly concentrated in the basal turn, whereas the middle turn showed only mild damage. Apex turn changes during aging have been reported (Jeng et al. 2020) (Wu et al. 2021) (Gleich et al. 2016). However, the significance of apical turn changes was not fully explored, and data on synaptic changes associated with aging and superimposed noise were not obtained. This issue should be addressed in future studies.

Another point that requires clarification is that the C57BL/6J mouse strain employed in this study carries a Cdh23 mutation that affects cadherin‐23 expression. This strain is widely used as an ARHL model and is characterized by ribbon synapses and progressive SGNs degeneration, leading to early‐onset progressive hearing loss (Peineau et al. 2021) (Mock et al. 2016) (Jeng et al. 2020). In other mouse strains, the correlation between ARHL and ribbon synapses loss is relatively weak (Jeng et al. 2020). Therefore, when superimposed noise damage occurs, synaptic and neuronal changes in C57BL/6J are more pronounced. Genetic variation in cadherin 23 may also contribute to susceptibility to NIHL in humans (Jiao et al. 2024).

Current quantitative data on CtBP2‐ and GluA2‐positive puncta indicate that most synaptic loss occurs during a 2‐h exposure period (Liberman et al. 2015). The blood elimination half‐life of brimonidine is 3 h, and its plasma protein‐binding rate is approximately 29% (Bin et al. 2021). Moreover, the median effective dose (ED50) and lethal dose (LD50) of intraperitoneally administered brimonidine in hypnotized mice were 75.7 and 379 mg/kg, respectively (Bin et al. 2021). Brimonidine was administered three times (1 mg/kg) 0.5 h before NE. Overall, the effective concentration of brimonidine was maintained throughout the NE duration, as evidenced by a decrease in synaptic damage. Therefore, the administration of an α_2_‐AR agonist may help alleviate neuronal damage in humans exposed to sound levels exceeding 100 dB, such as during otologic and neurotologic surgery. Dexmedetomidine, which is widely used for anesthesia induction (Douglas et al. 2025), may also confer protective effects on auditory nerves by reducing drill‐induced damage. Although anesthesia studies of brimonidine have already been conducted in animal experiments (Bin et al. 2021), further research is warranted. In our study, all mice exhibited sedative side effects after IP brimonidine injection but regained normal locomotor activity within 70 min. A previous ophthalmologic study reported that a higher dose (2 mg/kg) of IP brimonidine or a shorter treatment duration (2 days) produced neuroprotective effects, and sedation subsided spontaneously (Maciulaitiene et al. 2024).

A limitation of this study is that different brimonidine concentrations, administration methods, and administration schedules were not evaluated. Furthermore, the duration of the protective window against NE‐induced damage remains unclear.

Therefore, the use of brimonidine, a well‐established and safe drug, can shorten the research pipeline for its application as an inner‐ear neuroprotective agent. Its high selectivity and low lipid solubility reduce central side effects associated with blood–brain barrier penetration (Lee and Kim 2022) (Weber et al. 2007) (Robin and Burnstein 1998). Our animal experiments showed that IP injection is effective; however, to minimize systemic cardiovascular risks and build on clinical ophthalmology experience, local administration routes (such as tympanic cavity injection and ear drops) may be preferred. Future studies should clarify the pharmacokinetic profile of brimonidine in the inner ear.

According to a World Health Organization report, approximately 1.1 billion young people worldwide may be at risk of NIHL. Construction, mining, and manufacturing are the leading occupational sources of adult hearing loss; however, non‐occupational noise, such as improper use of personal audio systems, can also damage hearing (Murphy et al. 2018). Gates et al. observed that in cases of presumed cochlear damage resulting from prior NE, subsequent hearing loss worsened with age and extended beyond the initially affected frequency range (Gates et al. 2000). This observation suggests that the aging in ears with prior noise‐induced damage differs from aging in undamaged ears (Kujawa and Liberman 2006). Given the current high prevalence of noise exposure and aging, reports on the increased prevalence of NIHL in the early stages of life have exacerbated concerns about the long‐term effects of NE on young individuals. Our results demonstrate that although noise‐induced threshold shifts can initially recover, they mask gradually developing neuropathological changes, which are likely to have profound long‐term effects on auditory processing. This primary neurodegeneration can increase hearing difficulties in noisy environments and may contribute to perceptual abnormalities such as tinnitus and hyperacusis (Kohrman et al. 2020; Radziwon et al. 2019). Furthermore, mice in the brimonidine group had significantly better hearing than those in the NE group at old age. This highlights the importance of noise protection during youth, specifically to protect synapses and prevent underlying damage that could lead to severe hearing loss during old age.

## 5 Conclusions

Brimonidine protects auditory nerves primarily by modulating the glutamate–glutamine cycle in the synaptic cleft and safeguarding synapses, thereby preventing long‐term neuronal degeneration. Brimonidine is a promising therapeutic candidate for the prevention of NIHL. Overall, these findings emphasize the importance of implementing protective strategies against noise during youth to reduce the risk of severe hearing impairment in older age.

## Author Contributions

Haibo Wang and Lei Xu contributed to funding acquisition and project administration. Jing Cai designed the project. Na Zhang and Yongdong Song conducted the experiments. Chengfang Chen contributed to the revision of the manuscript, Ligang Kong and Yu Ai drafted the original manuscript, Yu Jin conducted the experiments and analysis. Qian Gao conducted the literature search. Daogong Zhang and Lei Xu provided some technical methods and revised the manuscript. All authors read and approved the final version of the manuscript.

## Funding

This work was supported by the National Natural Science Foundation of China, 82271176, 82271172, 82471174, 81700918, 82192861. Key R&D Program of Shandong Province, China (2025CXPT120, 2023CXPT038). Taishan Scholar Foundation of Shandong Province, tsqn202211357.

## Disclosure

Permission Statement: All figures and tables in the article are original and created by the authors.

## Ethics Statement

All research protocols were approved by the Animal Care Committee of Shandong University (No. ECAESDUSM 20,123,011). All experiments were conducted in accordance with ARRIVE guidelines and complied with the Guide for the Care and Use of Laboratory Animals of the National Institutes of Health. Only male C57BL/6J mice were used in this study; therefore, the sex‐based influence on the results was not evaluated.

## Conflicts of Interest

The authors declare no conflicts of interest.

## Supporting information


**Figure S1:** Application of brimonidine alone had no effect on hearing and synapses. (A) The application of brimonidine alone had no effect on ABR hearing. (B) Representative images showing the number of three‐turn synapses in the cochlea when brimonidine was applied alone. (C) The statistical results of B. Brimonidine had no effect on the number of synapses.
**Figure S2:** Detection results of CAP in each group at 80 dB. (A) Brimonidine significantly increased the amplitude of CAP at 12 kHz. (B) Brimonidine significantly shortened the latency of CAP at 32 kHz. *n* = 5. # *p* < 0.05 by one‐way analysis of variance (ANOVA) compared with NE (#); *p < 0.05, **p < 0.01, ***p < 0.001.
**Figure S3:** The number of synapses in the apex turn between three groups at 2 days post‐NE. No differences were observed in the synaptic counts among all groups at 2 days post‐NE.
**Figure S4:** Expression of α_2_‐ARs and glutamate metabolism‐related proteins in the inner ear at 2 days post‐exposure. (A–F) After NE, both α_2a_‐AR (A–C) and α_2c_‐AR (D–F) decreased, while brimonidine did not affect their expression. (G–H) Western blotting showed that vGluT1 expression was significantly downregulated in both the NE and brimonidine groups. (I–M) Glutamine synthetase (GS, I–K) and ERK (L–M) showed no differences in expression among the groups. *n* = 5. Scale bars: 100 μm for IF images. *p < 0.05, **p < 0.01, ***p < 0.001.
**Table S1:** Lists of antibodies for immunostainings and western blot.
**Table S2:** Quantitative PCR primers used in the experiments.

## Data Availability

Sequence data that support the findings of this study have been deposited in the GEO with the primary accession code GSE307393. The datasets used and/or analyzed during the current study are available from the corresponding author upon reasonable request.
