# Supplementary material for: Brimonidine Therapy for Protection From Noise‐Induced Hearing Loss
Source: Aging Cell. 2026 Apr 9;25(4):e70481. doi: 10.1111/acel.70481 (PMC13062936; doi:10.1111/acel.70481)
Supplement: Supplementary file 1 — Figure S1: Application of brimonidine alone had no effect on hearing and synapses. (A) The application of brimonidine alone had no effect on ABR hearing. (B) Representative images showing the number of three‐turn synapses in the cochlea when brimonidine was applied alone. (C) The statistical results of B. Brimonidine had no effect on the number of synapses. Figure S2: Detection results of CAP in each group at 80 dB. (A) Brimonidine significantly increased the amplitude of CAP at 12 kHz. (B) Brimonidine significantly shortened the latency of CAP at 32 kHz. n = 5. # p < 0.05 by one‐way analysis of variance (ANOVA) compared with NE (#); *p < 0.05, **p < 0.01, ***p < 0.001. Figure S3: The number of synapses in the apex turn between three groups at 2 days post‐NE. No differences were observed in the synaptic counts among all groups at 2 days post‐NE. Figure S4: Expression of α2‐ARs and glutamate metabolism‐related proteins in the inner ear at 2 days post‐exposure. (A–F) After NE, both α2a‐AR (A–C) and α2c‐AR (D–F) decreased, while brimonidine did not affect their expression. (G–H) Western blotting showed that vGluT1 expression was significantly downregulated in both the NE and brimonidine groups. (I–M) Glutamine synthetase (GS, I–K) and ERK (L–M) showed no differences in expression among the groups. n = 5. Scale bars: 100 μm for IF images. *p < 0.05, **p < 0.01, ***p < 0.001. Table S1: Lists of antibodies for immunostainings and western blot. Table S2: Quantitative PCR primers used in the experiments. [file ACEL-25-e70481-s001.doc]

**Supplementary Information:**


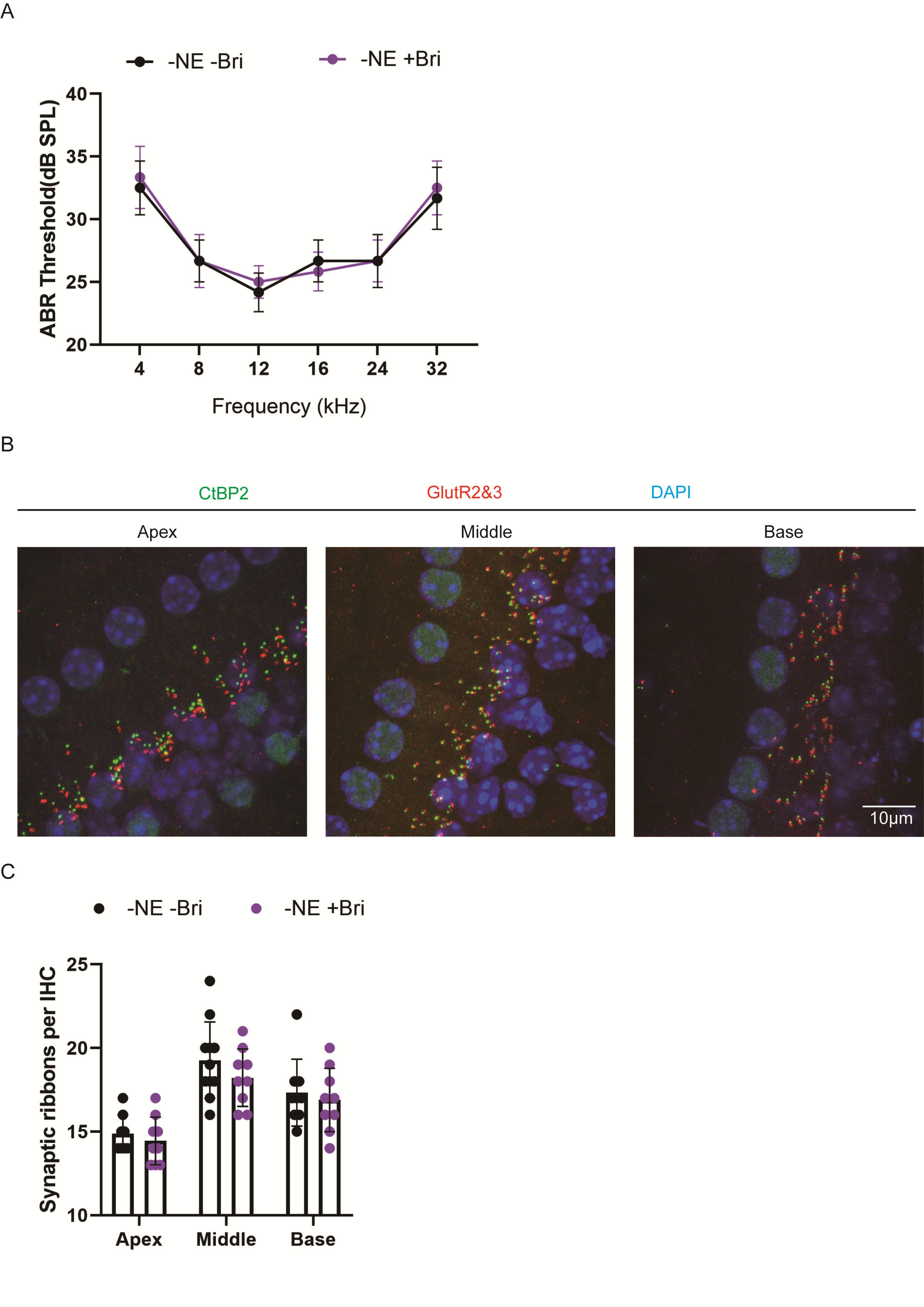


**Figure S1. Application of brimonidine alone had no effect on hearing and synapses.** A. The application of brimonidine alone had no effect on ABR hearing. B. Representative images showing the number of three-turn synapses in the cochlea when brimonidine was applied alone. C. The statistical results of B. Brimonidine had no effect on the number of synapses.


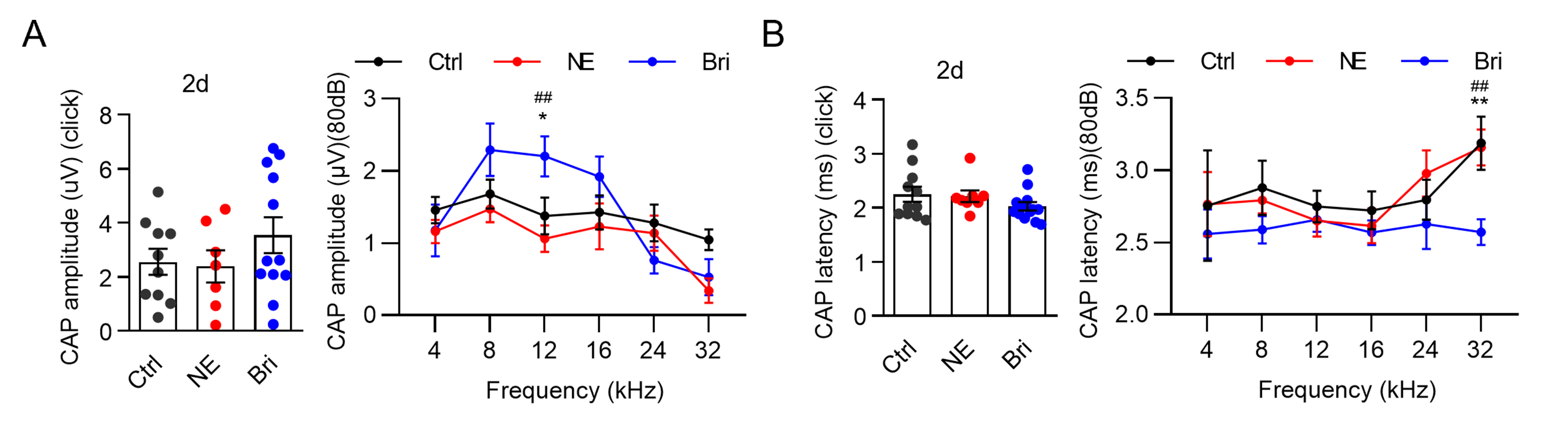


**Figure S2.** **Detection results of CAP in each group at 80 dB.**

A: Brimonidine significantly increased the amplitude of CAP at 12 kHz.

B: Brimonidine significantly shortened the latency of CAP at 32 kHz.

n =5. # p < 0.05 by one-way analysis of variance (ANOVA) compared with NE (#); *p < 0.05, **p < 0.01, ***p < 0.001.


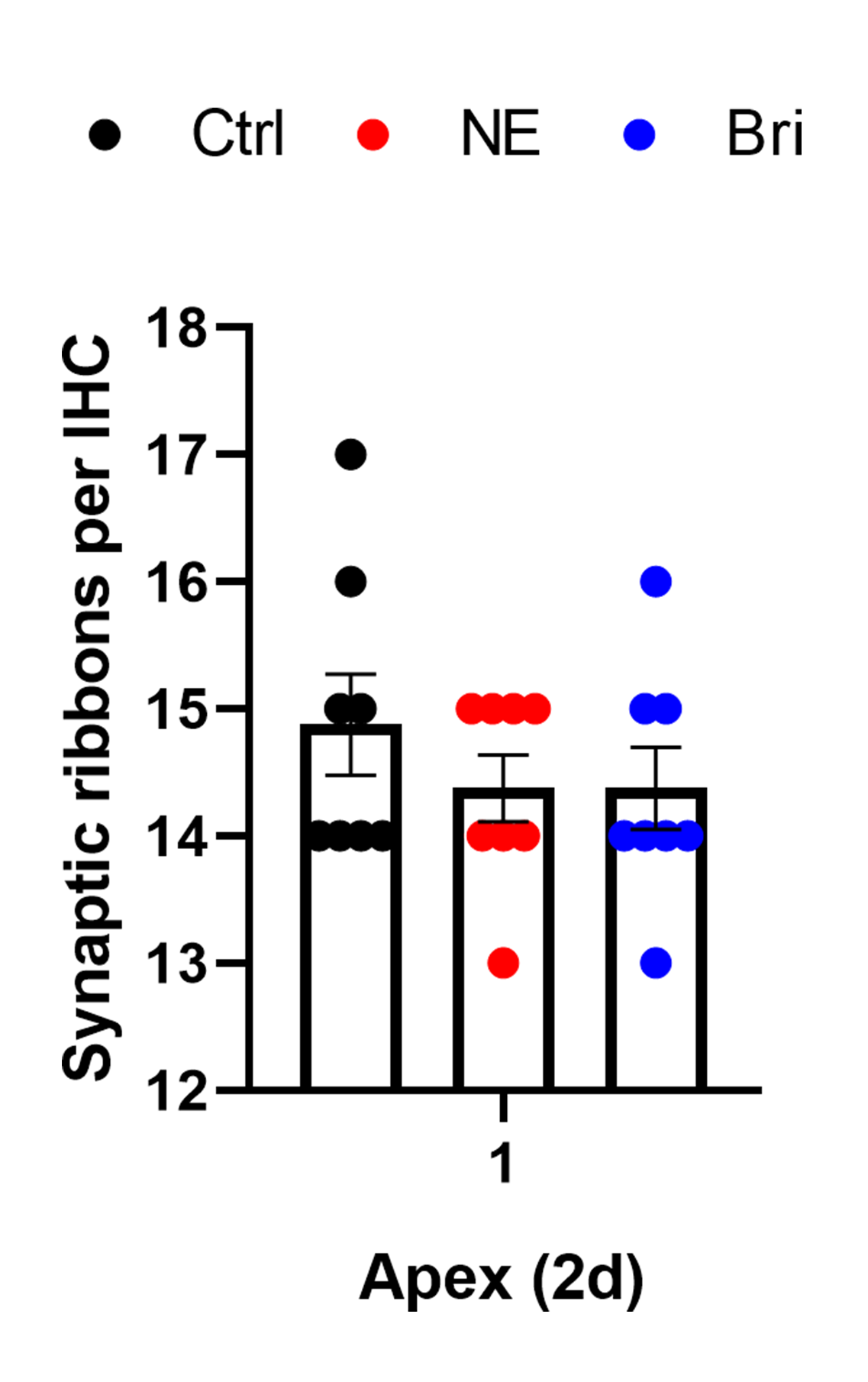


**Figure S3. The number of synapses in the apex turn between three groups at 2 days post-NE.**

No differences were observed in the synaptic counts among all groups at 2 days post-NE.


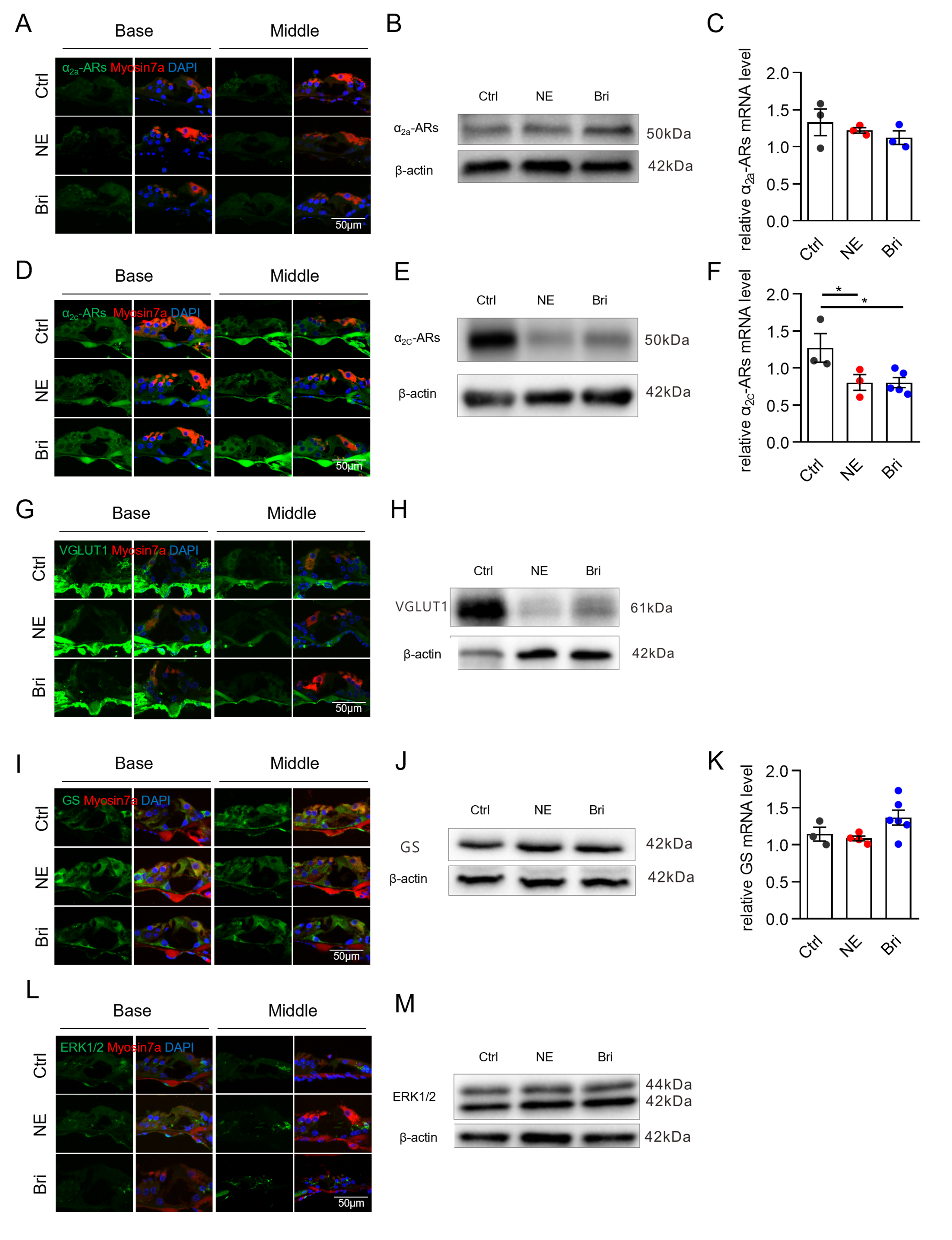


**Figure S4. Expression of** α_2_**-ARs and glutamate metabolism-related proteins in the inner ear at 2 days post-exposure.**

A–F After NE, both α_2b_-AR

A–C and α_2c_-AR

D–F decreased, while brimonidine did not affect their expression.

G–H Western blotting showed that vGluT1 expression was significantly downregulated in both the NE and brimonidine groups.

I–M Glutamine synthetase (GS)

I–K and ERK

L–M showed no differences in expression among the groups. n =5. Scale bars: 100 μm for IF images. *p < 0.05, **p < 0.01, ***p < 0.001.


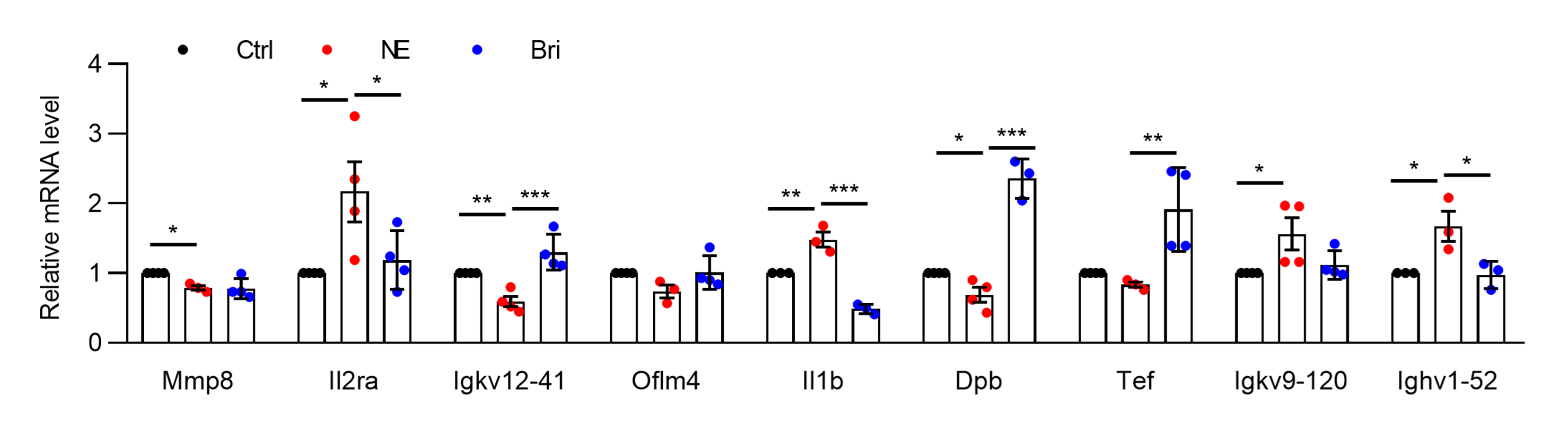


**Figure S5.** **Differentially expressed genes validation results.**

qPCR confirmed mRNA expression of DEGs.

Results were normalized to the GAPDH values. n =5. # p < 0.05 by one-way analysis of variance (ANOVA) compared with NE (#); *p < 0.05, **p < 0.01, ***p < 0.001.

Supplementary Table 1. Lists of antibodies for immunostainings and Western blot

| Antibodies | Source | Identification | Dilution |
| --- | --- | --- | --- |
| Myosin VIIa | Proteus BioSciences | Cat# 25-6790 | 1:1000 |
| Tuj1 | Abcam | Cat# ab78078 | 1:1000 |
| CtBP2 | Santa Cruz | Cat# sc-5967 | 1:200 |
| GlutR2 & 3 | Merck | Cat# AB1506 | 1:200 |
| α2a-AR | Thermo Fisher | Cat# PA1-048 | 1:500 |
| α2b-AR | Antibodies online | Cat# ABIN264463 | 1:500 |
| α2c-AR | Abcam | Cat# ab167433 | 1:200 |
| ERK | CST | Cat# 4695 | 1:200 |
| *p* -ERK | CST | Cat# 4370 | 1:500 |
| GLS | Abcam | Cat# ab93434 | 1:200 |
| VGLUT1 | Abcam | Cat# ab134283 | 1:200 |
| VGLUT2 | CST | Cat#71555 | 1:500 |
| VGLUT3 | Abcam | Cat# ab23977 | 1:200 |
| EAAT1 | Abcam | Cat# ab416 | 1:100 |
| GS | Abcam | Cat# ab73593 | 1:200 |
| Alexa Fluor 488 donkey anti-goat IgG (H+L) | Invitrogen | Cat# A11055; RRID:AB_2534102 | 1:1000 |
| Alexa Fluor 488 donkey anti-mouse IgG (H+L) | Invitrogen | Cat# A21202; RRID:AB_141607 | 1:1000 |
| Alexa Fluor 488 donkey anti-rabbit IgG (H+L) | Invitrogen | Cat# A21206; RRID:AB_2535792 | 1:1000 |
| Alexa Fluor 546 donkey anti-goat IgG (H+L) | Invitrogen | Cat# A11056; RRID:AB_2534103 | 1:1000 |
| Alexa Fluor 546 donkey anti-mouse IgG (H+L) | Invitrogen | Cat# A10036; RRID:AB_2534012 | 1:1000 |
| Alexa Fluor 546 donkey anti-rabbit IgG (H+L) | Invitrogen | Cat# A10040; RRID:AB_2534016 | 1:1000 |

**SUPPORTING INFORMATION**

**Supplementary Table 2. Quantitative PCR primers used in the experiments.**

| **Gene** | **Sequence** | |
| --- | --- | --- |
| ***a2a-AR*** | Forward (5′-3′) | CATCTCCTTCCCGCCACTCAT |
|  | Reverse (5′-3′) | GGAACCGATGGACGAGGAGAT |
| ***a2b-AR*** | Forward (5′-3′) | GCAATGCGCTGGTAATTCTGG |
|  | Reverse (5′-3′) | GTTGGCCAGAGAGAAAGGGATG |
| ***a2c-AR*** | Forward (5′-3′) | CACTGGTCATGCCCTTTTCTCT |
|  | Reverse (5′-3′) | TCCAGACTAATGGCACACAGGT |
| ***VGLUT*** | Forward (5′-3′) | TTGGGGCTGCGATACTGCTC |
|  | Reverse (5′-3′) | GCCAACCTACTCCTCTCCAAGG |
| ***EAAT1*** | Forward (5′-3′) | TCATCGTCCTGCCTCTCCTCTAC |
|  | Reverse (5′-3′) | GAGCACAAATCTGGTGATGCGT |
| ***GS*** | Forward (5′-3′) | GAACAGACGGCCACCCATTTG |
|  | Reverse (5′-3′) | GGCATAACCTCCGCATTTGTCC |
| ***Gls*** | Forward (5′-3′) | TGCAGAAGGACAAGAAAAGATACC |
|  | Reverse (5′-3′) | AGCATGACACCATCTGACGTT |
| ***Olfm4*** | Forward (5′-3′) | CCTTCAGTTCTGCCTTGGCTC |
|  | Reverse (5′-3′) | TATCCGGCAGGGAAACAGAACAC |
| ***Dbp*** | Forward (5′-3′) | CTCTTGCAGCTCCTCTTCCC |
|  | Reverse (5′-3′) | TAGCACCTCCACGGTGTCTG |
| ***Ighv1-52*** | Forward (5′-3′) | CTGGCTACACCTTCACCAGCTAC |
|  | Reverse (5′-3′) | GGCTGTGCTGGAGGATTTGTCTA |
| ***Tef*** | Forward (5′-3′) | GTCCCTGCTGGAGCATTCTT |
|  | Reverse (5′-3′) | GTGGAAGGACTCGCCATCGTAG |
| ***Igkv9-120*** | Forward (5′-3′) | TGCCCGACAAGTGAGACTGA |
|  | Reverse (5′-3′) | TGTTGACTGGCATTTGGGGGAT |
| ***Igkv12-41*** | Forward (5′-3′) | TCAGTCTCCAGCCTCCCTATC |
|  | Reverse (5′-3′) | CCACTGAACCTTGATGGCACAC |
| ***Il2ra*** | Forward (5′-3′) | CCAGCAACTCCCATGACAAATCG |
|  | Reverse (5′-3′) | TTCATGTTTCCAAGGAGGTGGC |
| ***Il1b*** | Forward (5′-3′) | GATGAAGGGCTGCTTCCAAACC |
|  | Reverse (5′-3′) | GGTGCTCATGTCCTCATCCTGG |
| ***Mmp8*** | Forward (5′-3′) | TTGCCCATGCCTTTCAACCAG |
|  | Reverse (5′-3′) | TGAGCAGCCACGAGAAATAGGT |
